# Editorial for the Genetics of Alzheimer’s Disease Special Issue: October 2021

**DOI:** 10.3390/genes12111794

**Published:** 2021-11-14

**Authors:** Laura Ibanez, Justin B. Miller

**Affiliations:** 1 Department of Psychiatry, Washington University in Saint Louis, St. Louis, MO 63110, USA; 2 Department of Neurology, Washington University in Saint Louis, St. Louis, MO 63110, USA; 3 Sanders-Brown Center on Aging, University of Kentucky, Lexington, KY 40536, USA

Alzheimer’s disease is a complex and multifactorial condition regulated by both genetics and lifestyle, which ultimately results in the accumulation of β-amyloid (Aβ) and tau proteins in the brain, loss of gray matter, and neuronal death. This Special Issue, entitled “Genetics of Alzheimer’s Disease,” focuses on genetic contributions to this debilitating disease that may lead to better targeted therapeutics and diagnostics. This issue contains six original research articles and two review papers that further our collective knowledge of Alzheimer’s disease etiology and genetic risk factors underlying the disease.

Ibanez, Cruchaga [1] present a snapshot of current work in Alzheimer’s disease genetics by contextualizing recent advances targeting immune responses from *CD33* and *TREM2* with more contemporary approaches investigating the amyloid cascade hypothesis. They conclude that the molecular mechanisms modulating Alzheimer’s disease pathogenesis should be considered broadly, and identifying additional genetic predisposition variants may lead to better treatment through early prediction and diagnosis. Shaw, Katsumata [2] found that limitations inherent with stringent multiple testing correction may mask the ability to detect Alzheimer’s disease risk from complex copy number variations and genes with coupled expression within immunomodulatory tyrosine-phosphorylated inhibitory motifs (ITIMs) or activation motifs (ITAMs). They show that protein quantitative trait loci associate with Alzheimer’s disease more frequently for genes encoding ITIM/ITAM family members than non-ITIM/ITAM genes. Additionally, mitochondrial dysfunction may play a role in Alzheimer’s disease, and differential transcription of the Translocase of Outer Mitochondria Membrane 40 (*TOMM40*) gene is associated with Alzheimer’s disease in postmortem brains [3].

Data mining is often used to identify novel disease-associated genetic risk factors for Alzheimer’s disease. Huckvale, Hodgman [4] describe challenges with data mining on data from the Alzheimer’s Disease Neuroimaging Initiative that may cause issues with various machine learning algorithms. They describe significant feature correlation, where >90% of all biomarkers are significantly correlated with at least one other biomarker in that dataset. They recommend removing highly correlated features before performing large-scale data analyses. Carpanini, Harwood [5] performed a targeted analysis of Alzheimer’s disease-associated genes within the complement system in the IGAP dataset, and they confirmed genetic associations for both *CLU* and *CR1*, but *C1S* was not significantly associated with Alzheimer’s disease. They conclude that larger genome-wide association datasets and long-read sequencing technologies may help better characterize the complement system genetic landscape and its role in Alzheimer’s disease risk.

Peripheral blood biomarkers offer a promising, non-intrusive mechanism for early Alzheimer’s disease diagnosis. Garofalo, Pandini [6] explore how non-coding RNA molecules longer than 200 nucleotides are differentially expressed in peripheral tissue of Alzheimer’s disease patients and may lead to noninvasive confirmatory targets or prognostic biomarkers. Specifically, plasma BACE1-AS levels are differentially expressed in the pre-symptomatic phase, indicating long non-coding RNA molecules may be a viable pre-symptomatic diagnostic target. Patel, Zhang [7] demonstrate that rare genetic variants significantly impact gene expression and gene co-expression in Alzheimer’s disease, indicating that set-based gene analyses are necessary to fully capture gene dynamics related to disease progression. Those pathway-level analyses confirmed substantial immune and inflammatory expression quantitative trait loci associated with Alzheimer’s disease, as suggested in the review published in this Special Issue [1]. An association between immune markers and Alzheimer’s disease is also proposed by van der Linden, De Witte [8], who drew a correlation between levels of blood cytokines and growth factors and Alzheimer’s disease genetic risk factors. They found that eight immune markers (three growth factors and five cytokines) were downregulated by Alzheimer’s disease genetic risk factors, while seven immune markers (five growth factors and three cytokines) were upregulated in the blood when Alzheimer’s disease genetic risk factors were present.

The articles included in this Special Issue cover a range of topics and provide comprehensive insights to direct future Alzheimer’s disease genetics research. The growing number of pathways, genes, proteins, and molecules that appear to be involved in Alzheimer’s disease is of special interest, which highlights the importance of analyzing Alzheimer’s disease as a systemic disease and not just a neurological disorder. Findings in the blood may serve as not only potential biomarkers or potential drug targets, but also help decipher the pathobiology of the disease. We anticipate that this Special Issue will help researchers search for additional genetic associations that will help refine our understanding of the etiology of this complex disease.

## Data Availability

Not applicable.
